# Dual Transcriptomic and Molecular Machine Learning Predicts all Major Clinical Forms of Drug Cardiotoxicity

**DOI:** 10.3389/fphar.2020.00639

**Published:** 2020-05-21

**Authors:** Polina Mamoshina, Alfonso Bueno-Orovio, Blanca Rodriguez

**Affiliations:** ^1^Department of Computer Science, University of Oxford, Oxford, United Kingdom; ^2^Insilico Medicine Hong Kong Ltd, Hong Kong, Hong Kong

**Keywords:** machine learning, cardiotoxic adverse effect, safety pharmacology, bioinformatics and computational biology, *in silico* analysis

## Abstract

Computational methods can increase productivity of drug discovery pipelines, through overcoming challenges such as cardiotoxicity identification. We demonstrate prediction and preservation of cardiotoxic relationships for six drug-induced cardiotoxicity types using a machine learning approach on a large collected and curated dataset of transcriptional and molecular profiles (1,131 drugs, 35% with known cardiotoxicities, and 9,933 samples). The algorithm generality is demonstrated through validation in an independent drug dataset, in addition to cross-validation. The best prediction attains an average accuracy of 79% in area under the curve (AUC) for safe versus risky drugs, across all six cardiotoxicity types on validation and 66% on the unseen set of drugs. Individual cardiotoxicities for specific drug types are also predicted with high accuracy, including cardiac disorder signs and symptoms for a previously unseen set of anti-inflammatory agents (AUC = 80%) and heart failures for an unseen set of anti-neoplastic agents (AUC = 76%). Besides, independent testing on transcriptional data from the Drug Toxicity Signature Generation Center (DToxS) produces similar results in terms of accuracy and shows an average AUC of 72% for previously seen drugs and 60% for unseen respectively. Given the ubiquitous manifestation of multiple drug adverse effects in every human organ, the methodology is expected to be applicable to additional tissue-specific side effects beyond cardiotoxicity.

## Introduction

Drug cardiotoxicity significantly limits the application of numerous therapies, and also slows down the drug research and development process (Cook et al., 2014; Onakpoya et al., 2016). As the attrition rate due to cardiotoxicity remains high, the need and importance of novel approaches capable of efficient safety testing has been widely emphasized, but not solved (Cook et al., 2014; Waring et al., 2015). Human-based approaches exploiting *in silico* methods have been postulated as the most promising alternative to costly animal experiments (Lawrence et al., 2008; Vicente et al., 2018), which frequently exhibit limited translation ability to human (Mak et al., 2014; Rodriguez et al., 2016).

In this regard, great progress has been made for example to evaluate the ability of *in silico* models to assess and predict the clinical risk of drug-induced arrhythmias (Lancaster and Sobie, 2016; Passini et al., 2017; Dutta et al., 2017). However, less attention has been paid to the prediction of other forms of drug-induced cardiotoxicity, such as cardiomyopathies, heart failure, myocardial ischemia or myocarditis (Mladěnka et al., 2018). Novel approaches are therefore needed to account for the wider spectrum of possible cardiovascular drug side effects beyond those mainly linked to adverse electrophysiological interactions.

Machine learning methods are gaining recognition in biological data analysis (Mamoshina et al., 2016; Lin and Lane, 2017; Lo et al., 2018). However, less than a handful of studies have addressed drug cardiotoxicity prediction beyond drug-induced arrhythmias. Huang and colleagues used protein-protein interactions to predict general cardiotoxicity for 578 drugs, using a support vector machine method (Huang et al., 2011). Using transcriptional profiles and fingerprints of 251 drugs, Wang et al. focused on prediction of gastrointestinal, liver and kidney toxicities, and myocardial infraction, a single form of cardiotoxicity, using an extra trees algorithm for multi-label classification (Wang et al., 2016). Messinis et al. (2018) developed a transcriptomic-based predictor of drug-induced cardiomyopathy with 31 drugs. Importantly, although all these studies reported relatively good accuracies (0.68 (Huang et al., 2011), 0.80 (Wang et al., 2016) and 1 (Messinis et al., 2018), respectively) under different cross-validation strategies (random split of samples or leave-one-drug out), none of them conducted an independent validation on drugs previously unseen by the trained model. This is crucial to ensure the translatability of proposed approaches to real-world applications, and thus an important limitation of previous work. In addition, none of the previous algorithms was developed to predict all major forms of drug cardiotoxicity.

Our hypothesis is that molecular and structural properties of drugs combined with their associated transcriptional changes in gene expression represent a suitable strategy to characterize their cardiac safety. The goal of this work is therefore to tackle four main challenges in cardiotoxicity prediction, namely prediction of six cardiotoxicity types, addressing the class imbalance problem, robust validation with independent datasets, and combination of transcriptional data and molecular descriptors. Our aim is to develop an independently-validated supervised machine-learning-based approach for the simultaneous prediction of all major forms of drug cardiotoxicity in human, using a substantial dataset of transcriptional and molecular descriptors compiled from diverse publicly-available data repositories. Our proposed approach specifically accounts for independent validation, the challenges of severe safety class imbalance and the preservation of relationships between different drug-induced cardiotoxicities. Addressing class imbalance is crucial as available datasets are usually heavily unbalanced (i.e., unequal distribution between drug types and/or cardiotoxic classes) (Banerjee et al., 2018), especially in large datasets curated automatically. This limits the generalization ability of data-driven methods, and in particular of unsupervised ones. In this work, we overcome these challenges by the application of supervised classifiers, as they generally demonstrate higher predictive performance on unbalanced biological data (Miller et al., 2008). Importantly, we demonstrate prediction of all six main forms of cardiotoxicity related to drug action in human. Through this work, we therefore significantly increase the domain of applicability and translation capabilities of machine-learning for cardiotoxicity prediction in preclinical drug evaluation. The findings and methodologies are expected to be generalizable to other organ-specific side effects.

## Materials and Methods

### Data Preparation

**Figure 1:**
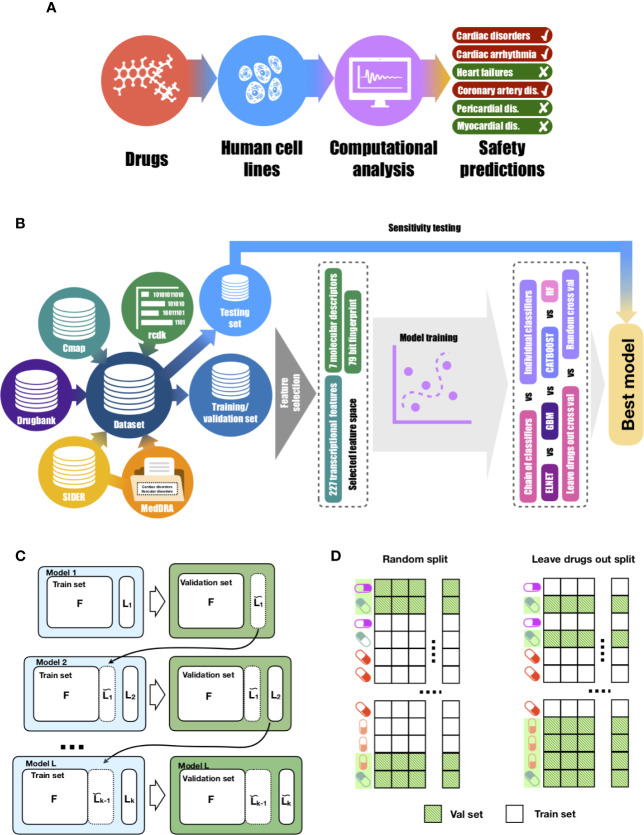
Proposed approach for drug-induced cardiotoxicty prediction. **(A)** *In silico* prediction of multiple forms of drug-induced cardiac adverse reactions. **(B)** The design of the proposed machine learning model builds on a comprehensive database of drugs linked to their transcriptional profiles, molecular descriptors, fingerprints and safety information. Our database was collated using several publicly-available knowledge and data repositories, as detailed in **Table 1**. The rcdk package was used to calculate the set of molecular descriptors and fingerprints. After collation, the whole dataset was split into training and testing sets of unique sets of drugs in each. Using training data, the set of the most predictive features was selected. Those features were later used to train multi-label models (chain of classifiers with nested stacking vs sets of individual classifier). Training was performed with two cross-validation strategies: random vs leave-drug-out. **(C)** Proposed chain of classifiers with nested stacking model for multi-label prediction of drug cardiac safety. Each classifier *L* in the chain is trained on a set of *F* features and the *L-1* labels predicted by the previous *L-1* classifiers. **(D)** Considered cross-validation approaches. Random cross-validation results in validation and testing sets having profiles of drugs from training sets, which leads to inflated performance on validation and low generalisation abilities on unseen test data compared to leave-drug-out cross-validation.

The first step was to curate a database of cardiotoxic and matching safe drugs (**Figure 1B** and **Table 1**), using diverse publicly-available knowledge and data repositories, including DrugBank (Wishart et al., 2008) (www.drugbank.com), Connectivity map Project (https://clue.io/cmap) (Subramanian et al., 2017), SIDER(Kuhn et al., 2016) (sideeffects.embl.de), MedDRA (https://bioportal.bioontology.org/ontologies/MEDDRA) and MESH (https://www.ncbi.nlm.nih.gov/mesh).

**Table 1:** Summary of databases and knowledge portals used in the study.

Name	Type of data	Link	Reference
DrugBank	Drug structure, list of targets	www.drugbank.com	(Wishart et al., 2008)
MedDRA	Side effect hierarchy	https://bioportal.bioontology.org/ontologies/MEDDRA	
SIDER	Drug safety information	sideeffects.embl.de	(Kuhn et al., 2016)
MESH	Pharmacological classes information	https://www.ncbi.nlm.nih.gov/mesh	
Connectivity map Project	Transcriptional profiles	https://clue.io/cmap	(Subramanian et al., 2017)
DToxS	Transcriptional profiles	https://martip03.u.hpc.mssm.edu/index.php	

Names and IDs were retrieved from the MedDRA dictionary for all cardiac (MedDRA ID 10007541) and vascular (MedDRA ID 10047065) disorders. Interactions of chemicals in the form of STITCH compound identifiers and their MedDRA terms of side effects were downloaded from the publicly available SIDER database. We used PubChem Compound Identifiers (CIDs) to match this list to the information on drug targets, drug status (‘approved', ‘investigational', etc.), and drug SMILES notations obtained from the Drugbank database. For drug target information, only experimentally verified interactions (such as inhibition, activation and intercalation between drugs and proteins or other molecules, like DNA) as provided by Drugbank were considered for this work. Compounds linked to the MedDRA term ‘cardiac disorders' were labeled as drugs with cardiotoxicity reports and were considered as ‘positive cases' in further model contraction. Compounds with same targets and no record of cardiac disorders in the database were considered as safe and as ‘negative cases' in further model contraction. Safe compounds were additionally filtered by their status, and only approved compounds currently on the market were used for analysis, resulting in 26 (out of 759) drugs being removed from the analysis. This was performed to prevent possible unreported toxicities in drugs considered safe but withdrawn from the market. The unsafe group includes both marketed and withdrawn drugs, including drugs withdrawn due to cardiac side effects. Information about therapeutic classes of drugs were collected from the MESH medical vocabulary.

In this work, we used the Connectivity map project as a source of gene expression cell responses to drugs, or drug transcriptional profiles. These were measured using an L1000 high-throughput profiling method. The L1000 fluorescent assay allows the detection and quantification of the expression of up to 978 landmark and 80 control transcripts simultaneously in each well of 384-well plate, where each well can contain a separate drug profile. This massive scale expression data is available in multiple levels starting from raw fluorescent intensity values from each well to replicate collapsed scores for drugs. In this work, we used well-established ‘core' human cell lines with drug transcriptional profiles available. We explored the provided transcriptional profiles (normalized across each scan plate) of the 977 ‘landmark genes' (Level 3a—NORM, as described in detail in https://clue.io/connectopedia/data_levels), for six cell lines (A549 and MCF7 for training and validation and PHH, SKB, SKM1, A673 for sensitivity testing), two incubation times (6 and 12 h), and multiple drug concentrations. To link the drug transcriptional profiles provided by the Connectivity map project to the information about their side effects, targets and status, we utilized the Chemical Translation Service (http://cts.fiehnlab.ucdavis.edu/) to match PubChem CIDs to their corresponding Broad IDs. In total, we collected a database of 1,131 drugs (fully analyzed for relationships across cardiotoxicity types), 357 of which had transcriptional profiles (used for prediction), with a total of 9,933 samples. Samples refer to independent transcriptional profiles of a drug at a given cell line, incubation time or concentration.

### Calculation of Molecular Descriptors and Fingerprints

We used the ‘rcdk' package (Guha et al., ) to calculate seven molecular descriptors widely used in drug property prediction (Dong et al., 2015; Zhang et al., 2016): molecular weight (MW), partition coefficient (XLogP), atomic polarisabilities (apol), topological polar surface area (TopoPSA), polar surface area expressed as a ratio to molecular size (tpsaEfficiency), Ghose-Crippen LogKow (ALogP) and molar refractivity (AMR). We also calculated the commonly used 79-bit ‘estate' fingerprint. These are widely used descriptors in drug property prediction and have been shown to characterize drug properties, including safety (Dong et al., 2015; Zhang et al., 2016).

### Selection of Transcriptional Features

In order to evaluate the predictive power of individual transcriptional profiles to cardiac safety prediction, following (Cai et al., 2018) two selection methods (correlation-based, wrapped-based) were considered. Correlation-based methods aim to identify transcriptional features (genes) highly correlated with each cardiotoxicity form. Wrapped-based methods use predictive models to score all combinations of feature subsets for each form of cardiotoxicity. As a correlation-based method, the ‘select.cfs' function from the Biocomb R package (Novoselova et al., 2018) was used, while the Boruta algorithm implemented in the Boruta R package (Lagani et al., 2017) was used as a wrapper-based method. This way, for each cardiotoxicity form, we identify two subsets of genes.

Cohen's Kappa scores (Cohen, 1960), calculated by the ‘Kappa.test' function from the fmsb R package, were used (i) to estimate the accuracy of classifiers, and (ii) to evaluate the similarities between vectors. The evaluation of similarities between vectors was applied to binary vectors of cardiotoxicity types, and between transcriptional features vectors selected using either correlation-based or wrapper-based methods, as described above. The Kappa scores are given by:

(1) $$\kappa =1-\;\frac{1-p_{o}}{1-p_{e}}$$

where *p_o_* is the relative observed agreement between two binary vectors, and *p_e_* is the expected agreement between predicted and actual values. Values smaller than 0 demonstrate poor agreement and values from 0.81 to 1 correspond to almost perfect agreement. To analyze whether the chosen genes were associated with the same or different biological functions, we also intersected the lists of determined genes with the Reactome database of pathways (Fabregat et al., 2018).

### Training, Validation and Testing Set Design

Models were trained on the expression values of relevant genes, seven molecular descriptors values and 79 fingerprint values, calculated as detailed above (340 features in total). We randomly split the entire drug dataset by protein targets (information obtained from the Drugbank) into unique training (291 drugs, 8,237 samples) and testing (66 drugs, 1,696 samples) sets. By such design, both datasets only overlap by a set of protein targets, but drugs on the testing phase are completely unseen during training, therefore facilitating preclinical translation to novel chemicals. This strategy was also enforced during model development, where for each cardiotoxicity label its respective training and validation sets were preserved completely non-overlapping by drugs with leave-drug-out cross-validation strategy (**Figure 1D**). We collapsed samples, so each drug profile referred to gene expression values for each individual drug with one cell line, incubation time and concentration. Models were trained on matrices size of 340 × 1,154 and tested on 340 × 746.

To benchmark the performance of models and select the best set of parameters, we used leave-drug-out cross-validation in contrast to random cross-validation (**Figure 1C**). This cross-validation strategy is crucial to accurately assess the performance of the model on unseen drugs, and therefore evaluate its translational potential into real-world practice. We performed the synthetic minority over-sampling technique (SMOTE) (Chawla et al., 2002), implemented in the DMwR R package (Torgo, 2013), on the training set in cross-validation to avoid overfitting at any stage. To determine the generalisation ability of methods for novel drugs, we assessed the best-performing models on the selected testing set of 66 unique drugs.

### Chain of Classifiers With Nested Stacking

To predict cardiovascular safety we employed a chain classifier with nested stacking, which takes into account label dependencies (**Figure 1C**). The chain of classifiers with nested stacking (Senge et al., 2019) is a model that receives a feature vector and maps it to a set of labels. Each classifier in the chain is trained on a set of features and the set of labels predicted by the previous classifiers. We used cardiotoxicity types as labels and gene expression values along with molecular descriptors and fingerprints as features. We used the following order of cardiotoxicity types obtained from MedDRA (see ‘Data Preparation'): ‘Vascular disorders', ‘Cardiac disorder signs and symptoms', ‘Cardiac arrhythmias', ‘Heart failure', ‘Coronary artery diseases', ‘Pericardial disorders' and ‘Myocardial disorders'. This order was based on the number of drugs related to those side effects. Because the first model will not receive the information from other cardiotoxicity types, we introduced vascular disorders (which is also related to cardiac disorders) as the first disorder for prediction, in order to minimize the effect of the first position for ‘Cardiac disorder sign and symptoms'. However, the accuracy of ‘Vascular disorder' prediction was not used in the evaluation of the model performance. This way, the chain of classifiers takes a set of features (transcriptional, molecular descriptors and fingerprints) and is tasked to predict whether the drug has cardiotoxicity reports (‘positive case') or not (‘negative case'), for six cardiotoxicity types.

To determine which algorithm accounts best for the observed data, we adapted several supervised binary classification algorithms widely used in bioinformatics: elastic net logistic regression (glmnet R library by Friedman et al. (Friedman et al., 2010)), random forest (ranger R library by Marvin et al. (Wright and Ziegler, 2017)), gradient boosting (gbm R library by Ridgeway et al. (Greenwell et al., 2007)) and categorical boosting (catboost R library by Prokhorenkova et al. (Prokhorenkova et al., 2018)). All models were optimized with Latin hypercube sampling of parameters (clhs R library by Roudier (Minasny and McBratney, 2006)) towards maximum Matthews correlation coefficient (MCC):

(2) $$MCC=\frac{\sum^{}tp\times \sum^{}tn-\sum^{}fp\times \sum^{}fn}{\sqrt{\left(\sum^{}tp+\sum^{}fp\right)\left(\sum^{}tp+\sum^{}fn\right)\left(\sum^{}tn+\sum^{}fp\right)\left(\sum^{}tn+\sum^{}fn\right)}}$$

where *tp* (true positives) and *tn* (true negatives) are the number of unsafe and safe compounds predicted correctly, respectively, and *fp* (false positives) and *fn* (false negatives) are the number of safe and unsafe drugs predicted wrongly, respectively. An MCC of 0 indicates that the prediction is not better than a random prediction, an MCC of 1 indicates perfect prediction or total agreement, and an MCC of −1 indicates total disagreement.

The optimized parameters are supplied in **Supplementary Table 4**. We trained models with five-fold cross-validation selected to leave drugs out to compensate for overfitting, and to receive more robust performance metrics. Once trained, to predict the cardiac safety of any unseen chemicals, the model only receives as inputs their transcriptional features, molecular descriptors, and fingerprints.

### Model Comparison

In this study, our proposed chain of classifiers with nested stacking for multi-label classification of drug cardiotoxicity is compared against a set of independent binary classifiers by cardiotoxicity types (meaning the drug has at least one side effect). We adapted the same four classification algorithms (elastic net logistic regression, random forest, gradient boosting and categorical boosting) for this task. We adjusted the set of hyperparameters and validation and tested models as described in the previous section. The optimized parameters for each model are supplied in **Supplementary Table 4**.

### Model Evaluation

In addition to MCC, the following metrics were used to evaluate model performance for each cardiotoxicity forms:

(3) $$Accuracy=\frac{\sum tp+\sum tn}{\sum tp+\sum tn+\sum fp+\sum fn},$$

where *tp* is a number of correctly predicted drugs with cardiotoxicity reports, *tn* is a number of correctly predicted drugs without cardiotoxicity reports, *fn* is a number of incorrectly predicted drugs with cardiotoxicity reports and *fp* is a number of incorrectly predicted without cardiotoxicity reports. Accuracy shows the ratio of correctly predicted drugs to a total number of drugs.

(4) $$F1=2\times \frac{precision\times recall}{precision+recall}$$

or F1 score, where

$$precision=\frac{\sum tp}{\sum tp+\sum fp}$$

and

$$recall=\frac{\sum tp}{\sum tp+\sum fn},$$

where *tp* is a number of correctly predicted drugs with cardiotoxicity reports, *tn* is a number of correctly predicted drugs without cardiotoxicity reports, *fn* is a number of incorrectly predicted drugs with cardiotoxicity reports and *fp* is a number of incorrectly predicted without cardiotoxicity reports. *Precision* equals the fraction of correctly predicted unsafe compounds in all compounds predicted as unsafe, whereas *recall* shows the sensitivity of a model and equals the fraction of correctly predicted unsafe compounds out of all real unsafe compounds.

### External Validation

As external validation data, we downloaded gene expression drug profiles from the Drug Toxicity Signature Generation Center (DToxS) website (https://martip03.u.hpc.mssm.edu/index.php). This website provides access to expression data of PromoCell cardiomyocytes (up to four lines) incubated with FDA approved drugs. In total, we obtained 1,338 samples, which were collapsed in the same manner as Connectivity map data, so each drug profile referred to gene expression values for one cell line, incubation time and concentration. As a result, models were tested on 654 profiles of 51 drugs, 18 of which were for the same drugs used for training and validation of models. We used ‘rcdk' package to calculate the same fingerprints and set of molecular descriptors as for the drug dataset that training and testing.

(5) $$AUC=\int_{-\infty }^{\infty }TPR(T)\left(-FPR'(T)dT\right)$$

or area under the receiver operating characteristic (ROC) curve, where TPR is the true positive rate (identical to recall) and FPR is the false positive rate. AUC measures the diagnostic ability of a predictor, where an AUC of 0.5 indicates that the prediction is not better than a random prediction, and an AUC of 1 indicates perfect prediction. The pROC R library by Robin et al. (2011) was used to calculate AUC values for the classifiers.

## Results

### Enriched Analysis and Prediction of Six Drug-Induced Cardiotoxicity Forms Using Transcriptional and Molecular Data

**Figure 1** describes the computational and dataset framework defined through this study. The six main drug-induced cardiotoxicity forms were identified from MedDRA as the focus for prediction: ‘Cardiac disorders signs and symptoms', ‘Cardiac arrhythmias', ‘Heart failure', ‘Coronary artery disease', ‘Pericardial disorders', and ‘Myocardium disorders' (**Figure 1A**).

Then, a large dataset of drugs was collected from diverse publicly-available data repositories (**Figure 1B**), including two sources of information: transcriptional profiles and derived molecular descriptors and fingerprints. This yielded information on 1,131 drugs, 357 of which had transcriptional profiles with a total of 9,933 samples. As a strategy for validation, these were split into unique training (291 drugs, 8,237 samples) and testing (66 drugs, 1,696 samples) sets. Training was blinded to drugs on the testing set, hence facilitating preclinical translation to novel unseen chemicals (**Figure 1C**).

Transcriptional and molecular descriptors for the drugs were used as inputs to the machine learning algorithms (**Figure 1D**). All machine-learning models were evaluated in performance on the blinded testing dataset, also considering two independent (random and leave-drug-out) cross-validation strategies (**Figure 1D**). Further details on study design are provided in *Methods*.

**Figure 2:**
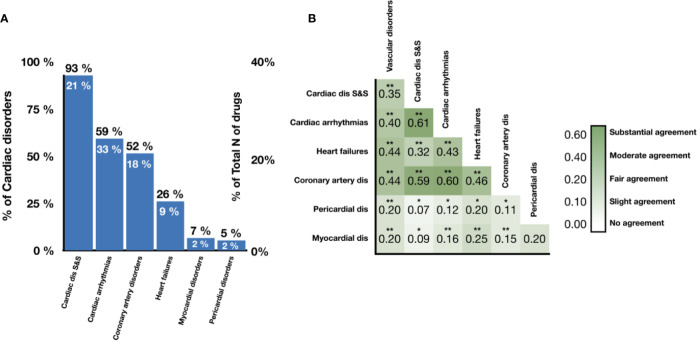
Prevalence and association of different cardiotoxicity forms in the drug database. **(A)** Proportion of drugs labeled as unsafe for given cardiotoxicity forms out of all unsafe drugs (numbers in black) and all drugs (numbers in white). **(B)** Levels of association between cardiotoxicity forms, measured in terms of Cohen's Kappa. Symbols represent statistical significance level by Z-test (*p <0.05, **p <0.01). ‘Cardiac dis S&S' is the MedDRA term for cardiac disorder signs and symptoms, ‘dis' is for disorders.

**Figure 2A** shows the number of drugs labeled as unsafe for the six groups of cardiotoxicity forms considered. Notably, 49% (46 out of 93) of antineoplastic drugs are reported to cause ‘cardiac disorders and signs and symptoms', indicating a high prevalence across cardiotoxicity forms and drug classes (**Supplementary Table 1**). On the other hand, 24% of CNS, 21% of CV, and 27% of antineoplastic agents produced cardiac arrhythmias, and 23% of CV and 25% of antineoplastic agents induced coronary artery disease (**Supplementary Table 1**). The prevalence of heart failure, myocardial disorders and pericardial disorders was lower for all drug classes, although still significant in some cases (for example, 14% of antineoplastic agents producing heart failure; **Supplementary Table 1**). We also evaluated the level of association between cardiotoxicity forms in terms of Cohen's Kappa (**Figure 2B**). ‘Cardiac arrhythmias' demonstrate a substantial association with both ‘cardiac disorder signs and symptoms' and ‘coronary artery disorders', with Cohen's Kappa values of 0.61 and 0.60 respectively. Interestingly, ‘heart failure' demonstrates a lower agreement with ‘cardiac disorder signs and symptoms' compared to ‘cardiac arrhythmias' and ‘coronary artery disorders' but higher agreement with ‘myocardial disorders'.

Based on the strong associations observed between drug-induced cardiotoxicity forms, we concluded that a prediction model should leverage dependance between side effects, and be able to re-use the information learned about the molecular basis of one side effect to better understand the molecular basis of others. This motivates our formulation of the cardiotoxicity prediction task as a multi-label classification problem, and our proposed machine-learning architecture as a chain of classifiers (**Figure 1D**), in order to preserve such relationships between drug-induced cardiotoxicity forms.

### Candidate Genes and Pathways Associated With Cardiotoxicity

**Figure 3:**
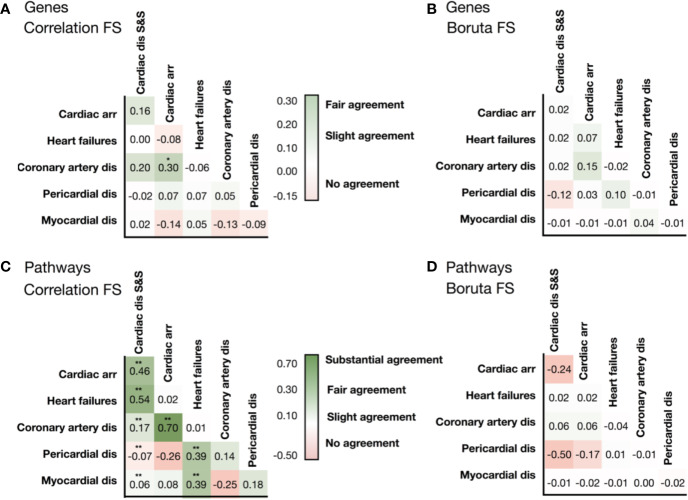
Similarities in the list of selected genes identified by **(A)** the correlation feature selection, and **(B)** the Boruta wrapper-based algorithm. Similarities in the list of pathways associated with genes identified by correlation-based feature selection **(C)**, and the wrapper-based algorithm **(D)**. Levels of association between cardiotoxicity forms are measured in terms of Cohen's Kappa. Symbols represent statistical significance level by Z-test (*p <0.05, **p <0.01). ‘Cardiac dis S&S' is the MedDRA term for cardiac disorder signs and symptoms, ‘dis' is for disorders.

We then evaluated whether the association between cardiotoxicity forms identified in **Figure 2** was also evident from transcriptional data, either in terms of genes or pathways. We hypothesized that transcriptional data would reveal underlying information on cardiotoxicity types when genetic pathways, rather than individual genes, are considered in the analysis. **Figures 3A, B** show the comparison of the gene vectors ranked as important by two feature selection methods for the different cardiotoxicity forms. Similarly, **Figures 3C, D** show this comparison in terms of the Reactome pathways to which those genes are related.

Analysis of the list of genes showed little to no intersection between them (**Supplementary Table 2**). Conversely, the consideration of pathways significantly improved the agreement (**Supplementary Table 3**). Gene and associated pathways for ‘cardiac disorders', ‘cardiac arrhythmias' and ‘heart failure' identified by both methods display moderate to high agreement, whereas for pericardial disorders no significant agreement was found. Interestingly, cardiac arrhythmias and coronary artery diseases show high similarity in both selected vectors of genes and pathways, and of label vectors of drugs.

Interestingly, G protein-coupled receptor transduction was selected as important by both methods for the prediction of cardiac disorder signs and symptoms (‘G alpha (q) signaling events' and ‘G alpha (s) signaling events') and heart failure (‘G-alpha (i) signaling events'). IGF1R and IGF1R-related signaling were also among the genes and Reactome terms selected by both methods for cardiac disorder signs and symptoms and for pericardial disorders. MAMLD1 gene, included in the Notch signaling pathways, was the only one ranked as important for predicting cardiac arrhythmias by both selection procedures. This further suggests association between different cardiotoxicity forms also at the feature level, which again motivates us to use a chain of classifiers to keep relations between cardiotoxicity forms during prediction.

### Machine Learning Prediction of Drug-Induced Cardiotoxicity Forms: The Importance of Leave-Drug-Out Cross-Validation

**Figure 4:**
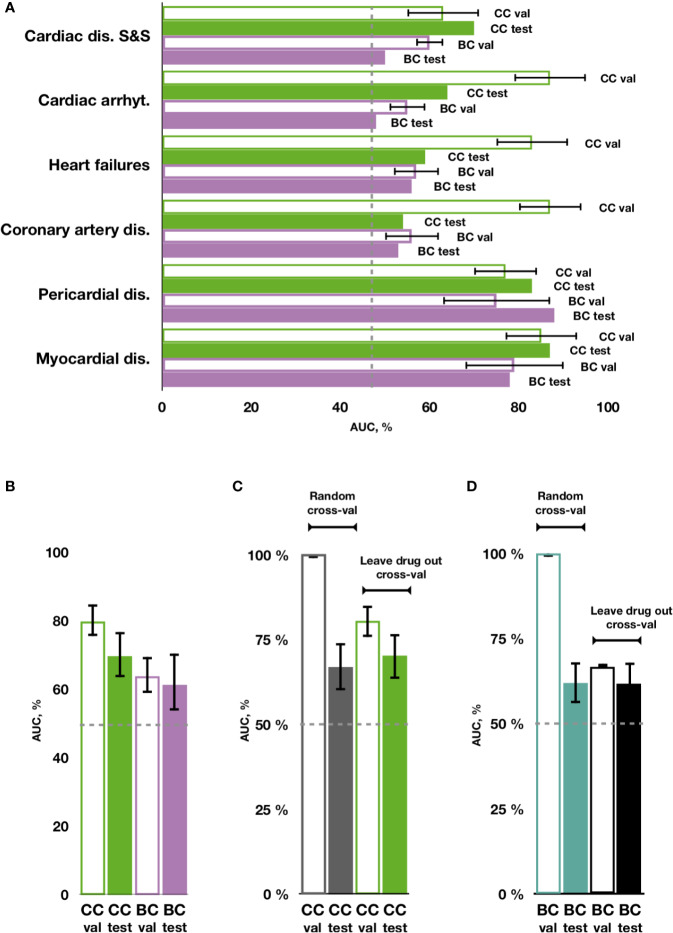
Prediction of cardiotoxicity forms using the chain of classifiers with nested stacking (CC) versus a set of binary classifiers (BC). **(A)** AUC of best performing CC and BC in safety drug prediction on validation (val) and testing sets (test) for each independent cardiac disorder. **(B)** Average AUC across all cardiac disorders for CC versus BC in validation and test sets. **(C, D)** Comparison between random vs leave-drug-out cross-validation strategies. Average performance across all labels is shown for the best performing chain of classifiers (CC) model, trained with leave-drug-out vs random cross-validation strategies, and for a set of independent binary classifiers (BC). Random cross-validation significantly inflates the accuracy of the trained models compared to leave-drug-out validation. Cardiac dis S&S': cardiac disorder signs and symptoms, ‘dis': disorders, ‘arrhyt': arrhythmias.

Using transcriptional and molecular features, all investigated forms of drug-induced cardiotoxicity were predicted with relatively good accuracy using the proposed chain of classifiers model with nested stacking trained with leave-drug-out cross-validation, and for all algorithms considered (elastic net logistic regression, gradient boosting, categorical boosting, and random forests). The best results were obtained for the chain of random forest classifiers, with an average AUC of 0.79 and an average MCC of 0.38 across all cardiotoxicity forms on validation, and 0.66 and 0.15 on testing (**Figure 4A**, **Table 2**, **Supplementary Figure 1** and **Supplementary Table 4**). The second best results were obtained with a chain of gradient boosting classifiers, with average AUC of 0.71 and average MCC of 0.24 on validation, and 0.66 and 0.15 on test set. The chain of categorical boosting classifiers showed average AUC of 0.77 and average MCC of 0.51 on validation, with average AUC of 0.65 and average MCC of 0.13 for testing. Finally, elastic net demonstrated the most modest performance among the trained set of chain classifiers, achieving an average AUC of 0.66 and average MCC of 0.16 on validation, with average AUC of 0.60 and average MCC of 0.11 for testing. Following these results, we selected a chain of random forest classifiers as the best model and evaluated its performance in detail, including validation on new cell types, external independent dataset and across cardiotoxicity types and pharmacological classes of drugs.

**Table 2:** The performance of multi-label classification models trained on transcriptional profiles and molecular descriptors and fingerprints of drugs on the validation and the testing set. The values are reported for Area under the receiver operating characteristic (ROC) curve (AUC; upper value), % and Matthews correlation (MCC; lower value).

Cardiotoxicity form	Set		Cardiac dis S&S	Cardiac arrhythmias	Heart failures	Coronary artery dis	Pericardial dis	Myocardial dis	Mean ± 0.5 SD
Leave drug out cross-validation strategy									
Chain of classifiers with nested stacking									
Dual feature set									
RF	Validation	AUC	63	88	79	91	64	88	79 ± 6
		MCC	0.21	0.50	0.29	0.68	0.13	0.46	0.38 ± 0.10
	*Testing*	*AUC*	*70*	*64*	*58*	*54*	*83*	*87*	***66 ± 9***
		*MCC*	*0.33*	*0.37*	*0.04*	*0.10*	*0.14*	*−0.08*	***0.15 ± 0.9***
ELNET	Validation	AUC	62	67	65	55	71	65	64 *±* 3
		MCC	0.20	0.23	0.19	*−*0.03	0.6	0.37	0.17 *±* 0.07
	*Testing*	*AUC*	*67*	*65*	*63*	*49*	*66*	*70*	*63 ± 4*
		*MCC*	*0.28*	*0.28*	*−0.08*	*−0.08*	*−0.03*	*0.01*	*0.06 ± 0.09*
GBM	Validation	AUC	67	87	74	90	60	86	77 *±* 6
		MCC	0.29	0.55	0.35	0.66	0.16	0.54	0.42 *±* 0.09
	*Testing*	*AUC*	*66*	*62*	*51*	*55*	*60*	*64*	*60 ± 3*
		*MCC*	*0.22*	*0.26*	*−0.08*	*0.03*	*0.12*	*−0.04*	*0.08 ± 0.07*
CATBOOST	Validation	AUC	64	84	64	88	65	53	70 *±* 7
		MCC	0.30	0.68	0.32	0.65	0.24	0.02	0.37 *±* 0.13
	*Testing*	*AUC*	*67*	*63*	*59*	*57*	*67*	*49*	*60 ± 3*
		*MCC*	*0.27*	*0.18*	*−0.07*	*0.09*	*0.35*	*0.0*	*0.14 ± 0.08*
Transcriptional features only									
RF	Validation	AUC	63	83	82	90	61	89	78 *±* 6
		MCC	0.24	0.47	0.4	0.66	*−*0.02	0.62	0.4 *±* 0.13
	*Testing*	*AUC*	*69*	*65*	*58*	*64*	*71*	*67*	*66 ± 2*
		*MCC*	*0.22*	*0.19*	*0*	*0.21*	*0.11*	*0.11*	*0.14 ± 0.04*
Descriptors and molecular fingerprints only									
RF	Validation	AUC	66	83	82	86	48	81	74 *±* 7
		MCC	0.24	0.55	0.37	0.51	*−*0.08	0.19	0.3 *±* 0.12
	*Testing*	*AUC*	63	72	42	56	62	58	59 *±* 5
		*MCC*	0.21	0.22	0.01	0.16	*−*0.08	*−*0.07	0.08 *±* 0.07
A set of independent binary classifiers									
RF	Validation	AUC	57	62	61	61	57	53	58 ± 2
		MCC	0.14	0.12	0.06	*−*0.03	0.0	0.17	0.08 *±* 0.04
	*Testing*	*AUC*	*64*	*63*	*59*	*59*	*45*	*58*	*58 ± 3*
		*MCC*	*0.21*	*0.18*	*−0.02*	*0.12*	*−0.01*	*−0.01*	*0.08 ± 0.05*
ELNET	Validation	AUC	60	58	61	55	72	64	62 ± 3
		MCC	0.12	0.15	0.10	*−*0.08	0.06	0.17	0.09 *±* 0.05
	*Testing*	*AUC*	*65*	*67*	*61*	*52*	*64*	*65*	*62 ± 3*
		*MCC*	*0.19*	*0.25*	*−0.03*	*−0.08*	*−0.04*	*−0.05*	*0.04 ± 0.07*
GBM	Validation	AUC	59	61	50	49	58	66	57 ± 3
		MCC	0.11	0.22	*−*0.02	*−*0.03	*−*0.14	0.37	0.08 ± 0.09
	*Testing*	*AUC*	*67*	*61*	*54*	*57*	*50*	*63*	*59 ± 3*
		*MCC*	*0.21*	*0.20*	*−0.02*	*0.04*	*−0.03*	*0.05*	*0.08 ± 0.05*
CATBOOST	Validation	AUC	62	60	61	56	54	70	60 ± 3
		MCC	0.23	0.26	0.20	0.28	0.07	0.37	0.24 ± 0.05
	*Testing*	*AUC*	*72*	*66*	*66*	*60*	*55*	*61*	*63 ± 3*
		*MCC*	*0.37*	*0.22*	*0.0*	*0.11*	*0.0*	*0.06*	*0.13 ± 0.07*
Random cross-validation strategy									
Chain of classifiers with nested stacking									
RF	Validation	AUC	92	96	93	95	94	92	**94 ± 1**
		MCC	0.70	0.77	0.53	0.72	0.44	0.26	**0.57 ± 0.10**
	*Testing*	*AUC*	*68*	*62*	*52*	*50*	*73*	*79*	*64 ± 6*
		*MCC*	*0.21*	*0.28*	*−0.09*	*−0.04*	*0.27*	*−0.07*	*0.09 ± 0.09*
A set of independent binary classifiers									
RF	Validation	AUC	90	88	74	83	86	77	**83 ± 3**
		MCC	0.67	0.61	0.25	0.57	0.56	0.16	**0.47 ± 0.11**
	*Testing*	*AUC*	*66*	*63*	*59*	*56*	*45*	*58*	*58 ± 4*
		*MCC*	*0.20*	*0.20*	*0.03*	*−0.09*	*−0.01*	*−0.01*	*0.05 ± 0.06*

Validation set and testing set performance are shown in the upper and the lower cells respectively and the best performance is shown in bold. RF is for random forest, ELNET is for elastic net logistic regression, GBM is for gradient boosting machines, CATBOOST is for categorical boosting. Cardiac dis S&S is for cardiac disorders signs and symptoms MedDRA term,dis is for disorders. Results for testing are shown in italics.

Importantly, cardiotoxicity types predicted with the best performing chain of classifiers model kept similar relationships to the ones shown in the original data (**Figure 2** and **Supplementary Figure 2**). This was particularly clear for the predominant associations between cardiac disease signs and symptoms, cardiac arrhythmias and coronary artery disease.

With individual binary predictors, the best set of random forest classifiers only obtained an average AUC of 0.67 and an average MCC of 0.08 across cardiotoxicity types on validation, with an average AUC of 0.62 and average MCC of 0.16 on testing (**Figure 4A**, **Table 2**, **Supplementary Figure 1** and **Supplementary Table 4**). Therefore, while cardiotoxicy types were predicted with different accuracies, the inclusion of information about other cardiotoxicities improved prediction accuracy for all cardiotoxicity types (**Figure 4B**). This was also observed on five different partitions of the entire dataset (**Supplementary Figure 3**), where the chain of classifiers outperformed the sets of individual binary classifiers. In line with that, the exclusion of cardiotoxicity forms resulted in a decreased prediction accuracy of sequential labels (**Supplementary Table 4**).

On the contrary to leave-drug-out, in the case of random cross-validation, samples using the same drugs may be present in training and testing datasets, and thus predictors learn associations between individual drugs and their safety rather than general features related to cardiotoxicity forms. This may produce unrealistically high results, indeed overestimating the accuracy of prediction.

To investigate these aspects in further detail, we compared predictions with our proposed chain of random forest classifiers with nested stacking and a set of independent random forest classifiers, both trained with either leave-drug-out or random cross-validation strategies. When validated and optimized with random cross-validation, both models demonstrate almost perfect accuracy on validation (averages for all cardiotoxicity types: AUC = 1, MCC = 0.95 for chain of classifiers; AUC = 1, MCC = 0.97 for independent predictors; **Figures 4C, D**, **Table 2** and **Supplementary Table 4**). Although apparently outperforming the models trained with leave-drug-out cross-validation, models trained with random cross-validation were however less accurate when predicting cardiotoxicity types of previously unseen drugs (**Figures 4C, D**, **Table 2** and **Supplementary Table 4**). Quantitatively, the chain of classifiers trained and optimized with a random cross-validation strategy exhibited an AUC of 0.67 and MCC of 0.13, compared to an AUC of 0.70 and MCC of 0.16 for leave-drug-out cross-validation (average values for all cardiotoxicity types). Similarly, a set of individual classifiers with random cross-validation showed an AUC of 0.62 and MCC of 0.09, compared to an AUC of 0.62 and MCC of 0.15 in the case of leave-drug-out cross-validation.

**Figure 5:**
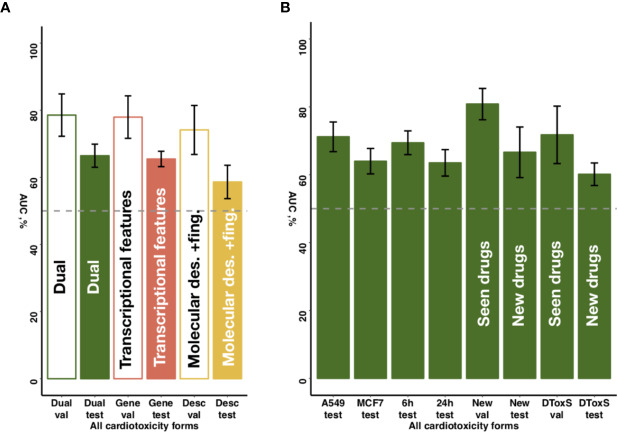
Sensitivity of predictive accuracy to feature types, cell lines and incubation times. **(A)** Merging of molecular descriptors and fingerprints and transcriptional features results in increased average performance across all drug classes. **(B)** The best performing model demonstrates similar predictive accuracy across different cell lines (A549, MFC7) and incubation times (6 and 24 h) incubation times. For new cell lines (PHH, SKB, SKM1, A673), the model discriminates more accurately seen drugs with unseen cell lines (‘New val') than unseen drugs (‘New test'). For new data type (DToxS), the model also discriminates more accurately seen drugs (‘DToxS val') than unseen drugs (‘DToxS test').

To further explore the predictive value of individual feature sets, we compared for the entire dataset the proposed chain of random forest classifiers with nested stacking separately trained on each feature set. Models trained on both feature types outperformed models trained only on one feature, showing on validation an AUC of 0.80 for both feature types vs an AUC of 0.62 for molecular descriptors and fingerprints, or an AUC of 0.76 for transcriptional features only (**Figure 5A**, **Table 2**, **Supplementary Table 4**). Similarly, the dual transcriptomic and molecular classifier is more accurate on testing. The model trained only on molecular descriptors and fingerprints achieves 0.65 AUC, being 0.66 AUC when trained only for transcriptional features.

### External Validation

To further investigate the predictive power of the developed predictor and to assess its performance on different source of transcriptional data, we additionally analyzed the DToxS dataset (https://martip03.u.hpc.mssm.edu/index.php).

Notably, while DToxS provides gene expression data measured with a different technique (RNAseq) and with different cell lines (PromoCell cardiomyocytes), the proposed chain of random forest classifiers with nested stacking still achieved good accuracy when discriminating previously seen drugs (average AUC of 72%, **Figure 5B**). The accuracy partially drops when predicting cardiotoxicity forms for unseen drugs (average AUC of 60%, **Figure 5B**). Interestingly, while the model trained on molecular descriptors and fingerprints only shows superior accuracy in predicting seen drugs (average AUC of 90%, **Supplementary Table 4**), the dual transcriptomic and molecular classifier is still more accurate on testing for unseen drugs. For example, it is able to differentiate safe from drugs with reports of cardiac arrhythmias with an AUC of 70% (vs 58% for molecular descriptors and fingerprints, **Supplementary Table 4**). The transcriptional feature only model shows less accuracy when tested on new data, with an average AUC for all labels of 57% (**Supplementary Table 4**). However, this model is more accurate in predicting cardiac disorder signs and symptoms (AUC of 65% vs 56% and 52%, for dual and molecular descriptors and fingerprints models, respectively; **Supplementary Table 4**).

### Predictive Accuracy Across Drug Classes and Cardiotoxicity Forms

**Figure 6:**
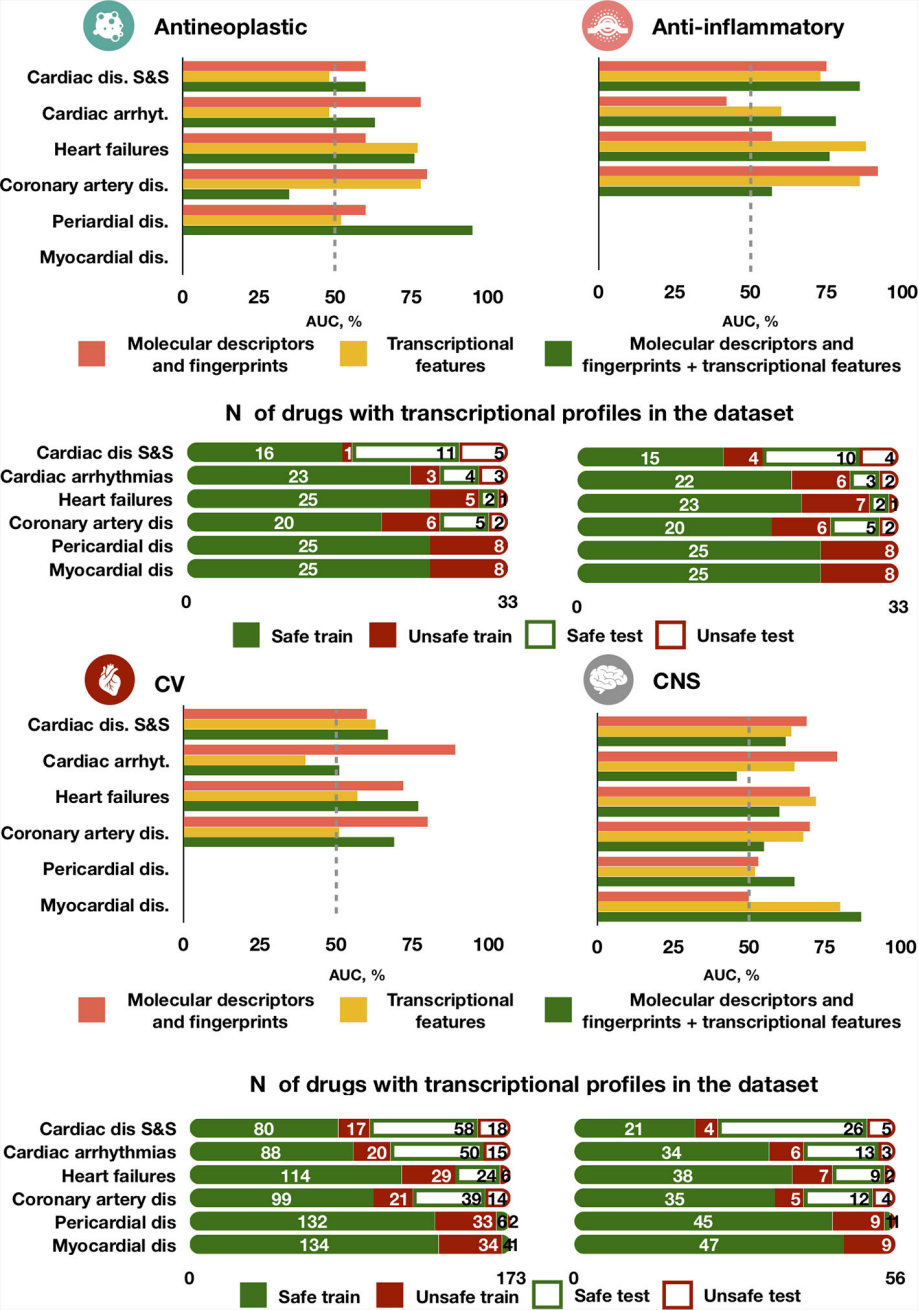
Predictive accuracy across drug classes, cardiotoxicity forms and feature types. The best performing model trained on either molecular descriptors and fingerprints, transcriptional features or both demonstrates different performance in predicting types of agents. For each drug class, the top bar plot shows the AUC of the best predictor and the bottom bar plot displays the number of safe and unsafe drugs. CV for cardiovascular agents, CNS for Central nervous system agents.

The best model (chain of random forest classifiers with nested stacking) demonstrated different predictive accuracy across cardiotoxicity forms and drug classes (**Figure 6** and **Supplementary Table 4**). For example, for antineoplastic drugs, the best model predicted ‘pericardial disorders' more accurately (**Figure 6**; AUC = 0.95) than the average across all drugs, also achieving high accuracy in the prediction of ‘heart failure' (AUC = 0.76). ‘Cardiac disorder signs and symptoms', ‘cardiac arrhythmias' and ‘heart failure' were also more accurately predicted in the case of anti-inflammatory drugs compared to other cardiotoxicity types (**Figure 6**; AUCs of 0.86, 0.78 and 0.76, respectively).

High accuracy was also achieved in the prediction of ‘myocardial disorders' by cardiovascular agents, as well as for ‘heart failure' induced by central nervous system agents (both with AUC = 0.77, **Figure 6**). On the contrary, predictions for ‘cardiac arrhythmias' were close or worse than random guessing for cardiovascular and central nervous system agents (AUCs of 0.51 and 0.46, respectively) and for cardiovascular agents (AUC = 0.51). Greater error in the prediction of ‘coronary artery disorders' was found for antineoplastic agents (AUC = 0.35). Some of these cases of limited performance may be partially explained by a small frequency of side effects for specific drug types. For example, only 16 out of 56 total cardiovascular agents in our dataset are known to cause cardiac arrhythmias and coronary artery diseases (**Figure 6**). However, for antineoplastic drugs the frequency of coronary artery disease (7/33) is the same than for cardiac arrhythmias (7/33) and bigger than for heart failure (3/33), but the predictor performs better in predicting the latter two than coronary artery disease, indicating a possible dependency on feature types.

Interestingly, the model trained individually on molecular descriptors and fingerprints demonstrated higher accuracy than the transcriptomic or dual predictors in safety predictions of ‘cardiac arrhythmias' for cardiovascular, antineoplastic and central nervous system agents, but not for anti-inflammatory agents (**Figure 6**). For central nervous system agents, in general, the molecular descriptor-based predictor is more accurate. Notably, for ‘coronary artery disorders', a combination of the two feature types leads to a decrease in accuracy compared to individual feature set predictors.

For interpretability of these prediction differences, we analyzed the feature space in testing against the best model predictions when trained on either individual or combined feature types (**Supplementary Table 5** and **Supplementary Figures 4–8**). Different transcriptional features and molecular descriptors were associated with the accuracy of prediction. For instance, drug samples with higher expression values of Alpha-Synuclein (SNCA) or Heat Shock Protein 8 (HSPA8) genes were more often predicted incorrectly by all three predictors (**Supplementary Figures 4** and **5**). Drugs with a higher number of atomic bonds (nAtomBond) (**Supplementary Figure 6**) were classified less correctly when training on the combined feature set or only on molecular descriptors. The same was observed for drugs with higher values of polar surface area to molecular size ratio (tpsaEffiency) or topological polar surface area based on fragment contributions (TopoPSA) (**Supplementary Figures 7** and **8**).

## Discussion

This study presents the first machine learning approach capable of predicting six forms of drug-induced cardiotoxicity from both gene expression and molecular descriptors data. Importantly, the algorithm (based on a chain of classifiers) is specifically developed to incorporate relationships between cardiotoxicity forms, identified in our data analysis, and to tackle class imbalance between cardiotoxic and safe drugs. We demonstrate high accuracy with the strictest validation strategy using drug datasets not used in training, and its importance compared to random cross-validation on samples. A further specific contribution of this study is the large comprehensive dataset of 1,131 drugs curated and collected from publicly available resources. This can provide a useful benchmark for future studies. Thus, we propose a novel and robust solution for preclinical drug safety testing that can potentially be expanded to other organs' toxicities.

To implement this solution, we first collected and analyzed a large dataset of 1,131 drugs from publicly available databases. They include both safe and cardiotoxic drugs, 357 of which with cellular transcriptional profiles and a total of 9,933 samples available. Secondly, we proposed and implemented a chain of classifiers with nested stacking approach that classifies drugs by their risk, able to relatively accurately predict up to six forms of cardiotoxicity. Our method achieves a 0.80 average AUC across all cardiotoxicity types on a leave-drug-out cross-validation strategy on 291 drugs (8,237 samples). Further validation of the method on new and previously unseen 66 drugs (1,696 samples) with multiple mechanisms of action demonstrated that the proposed model holds high generalisation abilities compared to sets of individual classifiers. Models trained with leave-drug-out cross-validation were able to discriminate between safe and unsafe drugs with a 0.70 average AUC across all cardiotoxicity types. The model demonstrated higher accuracy for specific adverse drug effects and type of agents, and in particular for pericardial disease, cardiac disease and symptoms, heart failure and myocardial disease for antineoplastic, anti-inflammatory, cardiovascular and central nervous system agents, respectively. These results suggest the translational potential of the proposed approach towards applications in a pre-clinical context.

The combined dataset collected in this study demonstrated associations between forms of drug-induced cardiac complications, which are in agreement with the known literature on cardiac comorbidities. Clinical reports have evidenced a significant association between heart failure and other cardiovascular comorbidities, such as atrial fibrillation, ischemic heart disease and arrhythmias (Lawson et al., 2018; Kendir et al., 2018). Patients with a history of coronary heart disorders have been also shown to have a higher incidence of atrial fibrillation, one of the most common forms of cardiac arrhythmias (Naser et al., 2017). In our work, coronary artery disorders, which include ischemia and myocardial infarction, demonstrated a significant similarity to cardiac arrhythmias in terms of the drugs they are related to. We showed the same for heart failure and cardiac disorders, which displayed a moderate and substantial agreement to cardiac arrhythmias.

Notably, the list of genes identified as most important features (obtained by using two completely distinct feature selection methods while counting distinct genes for each label) demonstrates a significant amount of intersection at the level of associated pathways. For example, pathways related to Notch signaling were ranked as important for cardiac arrhythmia prediction by both methods. Previous evidence has shown the importance of Notch signaling in heart development and cardiac disease, including malignant congenital arrhythmias (D'Amato et al., 2016). IGF signaling and IGF1R were also selected consentaneously by both algorithms for cardiac disorder signs and symptoms and pericardial disorders, evidencing their key role in heart tissue functioning (Troncoso et al., 2014). In spite of being profiled using cancer cell lines, the selected features seem biologically relevant to cardiotoxicity, given their human origin. This emphasizes the potential benefits of using the combined approach for feature selection. At the same time, further detailed investigation of the feature importance list could help evaluate the proposed genes as possible therapeutic targets for cardiovascular therapies.

Our method takes advantage of the chain of classifiers approach. This approach significantly outperformed binary classification approaches that treat each label independently, with an improvement of 12.9% in terms of AUC (from AUC of 0.62 to 0.70). This highlights the importance of incorporating information about related adverse reactions in predicting drug safety. While demonstrating good generalisation abilities on unseen data, the model showed different performance across cardiotoxicity types depending on the type of agents. Cardiac arrhythmia-related safety was predicted more accurately for cancer and anti-inflammatory agents than for cardiovascular and central nervous system agents. This might be improved by the introduction of information more relevant to specific mechanisms of arrhythmogenesis.

The accumulated body of evidence suggests that gene expression signatures alone could also be used as a biomarker of cell response to drugs (Aliper et al., 2016; Xie et al., 2018). Our results, in line with previous studies (Wang et al., 2016), demonstrate that coupling of transcriptional profiles and molecular descriptors indeed improves the predictive power of algorithms. Thus, the combination of both feature types indeed increases the mean accuracy of a chain of classifiers by 8% (AUC of 0.65 to 0.70).

Previous research (Wang et al., 2016; Messinis et al., 2018) reported good accuracy in the prediction of multiple adverse reactions and myocardial infarction. However, such approaches were evaluated using drugs included in the training, rather than in an independent dataset as in our study, and neglected existing dependencies between various forms of cardiotoxicity, as demonstrated here. Indeed, our findings suggest that the retention of the information about cardiotoxicity types dependencies results in greater accuracy (**Figures 4A, B**). At the same time, we show that models trained with random cross-validation may display a significantly inflated performance on validation (**Figures 4C, D**), however becoming less accurate when predicting previously unseen drugs. In general, a leave-drug-out cross-validation strategy demonstrated a more robust performance compared to random cross-validation, the latter evidencing inflated accuracy metrics, which in turn may complicate model optimisation and overstate their expected generalization ability. Our proposed chain of classifiers model with nested stacking has indeed better generalization for multi-label predictions than previous models such as that by Wang and colleagues (Wang et al., 2016), and demonstrates a superior performance compared to models based on sets of individual classifiers.

Our model can predict both acute (cardiac arrhythmias) and chronic (coronary artery disorders and heart failures) effects of drugs, based on clinical human responses collected *via* SIDER. Chronic drug-induced cardiac changes are often irreversible and their effect is delayed. Therefore their prediction poses a challenge, as they require long-term animal experimentation and the drug effect on the animal cardiac system is highly variable and hard to translate into the human clinic (Lamberti et al., 2014). Another key advantage of our approach is the use of perturbation databases such as LINCS and Connectivity map. They constitute great resources for preclinical applications (Musa et al., 2018) and even have been used extensively to identify novel drug candidates that confirmed their effectiveness experimentally (Han et al., 2018). Providing a cheaper alternative to animal models, computational methods that integrate transcriptional responses of drugs and their molecular characteristics, such as proposed, can be used prior to animal experiments identifying drug cardiotoxicity early in the pre-clinical phase.

Using transcriptional signatures, molecular descriptors and fingerprints, the general methodology proposed in this study could be further applied to other tissue-specific side effects and organs. We presume that our approach can be also extended to other areas including drug target prediction, where information about the multi-label properties of drugs or multi-target properties plays a vital role (Ramsay et al., 2018).

A current limitation of the database used in this study is the absence of isomers (drugs with similar chemical structure, and hence similar molecular descriptors and fingerprints) with different safety profiles. While we expect such chemicals to have different transcriptional profiles, and therefore to be discriminated by their transcriptional features, further analysis is required to test this hypothesis. Our model demonstrated good generalisation properties under completely new cell lines (**Figure 5B**). However, cross-platform differences in the acquisition of transcriptome data pose additional challenges for future use, and validation on an external dataset of RNAseq expression samples showed a moderate loss of prediction accuracy (**Figure 5B**). In addition, a limiting factor in the number of drugs used in this study was the availability of drug transcriptional profiles (rather than information on drug-induced cardiotoxicities), and the release of additional datasets would be a valuable resource for future studies. This study is also constrained by the feature space explored, with only 971 of landmark genes analyzed, seven molecular descriptors, and one type of fingerprints used for model contraction. Inclusion of information about expression values of other genes or more comprehensive descriptors and fingerprints might increase the predictive power of models and bring more insights. Machine-learning algorithms, however, are known to be limited in their ability to provide an interpretation of learnt associations. Mechanistic models coupled with machine-learning-based approaches represent an alternative attractive approach, with the potential to shed light on aspects of the underlying cardiotoxicity mechanisms (Lancaster and Sobie, 2016). Whereas these aspects fall beyond the goal of our study in presenting our proposed approach, they deserve future consideration in order to refine its predictive power, so it does comparison against other multi-target machine-learning algorithms.

## Data Availability Statement

The full curated dataset, together with a dedicated R package for cardiotoxicity prediction, are freely available upon request.

## Author Contributions

PM planned study, performed analysis and prepared the manuscript. AB-O and BR planned the study and prepared the manuscript.

## Funding

The authors are financially supported by a BHF Intermediate Basic Science Research Fellowship (to AB-O, FS/17/22/32644), a Wellcome Trust Senior Research Fellowship in Basic Biomedical Sciences (to BR, 100246/Z/12/Z and 214290/Z/18/Z) and the European Union's Horizon 2020 research and innovation programme under Grant Agreement No. 675451 and 823712 (CompBioMed project). BR and AB-O also acknowledge additional support from an Impact for Infrastructure Award of the National Centre for the Replacement, Refinement & Reduction of Animals in Research (NC/P001076/1). PM is funded by Insilico Medicine Hong Kong Ltd. The funders did not have a role in the study design, data collection, and decision to publish.

## Conflict of Interest

PM is associated with Insilico Medicine Hong Kong Ltd, engaged in drug discovery research.

The remaining authors declare that the research was conducted in the absence of any commercial or financial relationships that could be construed as a potential conflict of interest.
