# Supplementary material for: Dual Transcriptomic and Molecular Machine Learning Predicts all Major Clinical Forms of Drug Cardiotoxicity
Source: Front Pharmacol. 2020 May 21;11:639. doi: 10.3389/fphar.2020.00639 (PMC7253645; doi:10.3389/fphar.2020.00639)

Dual transcriptomic and molecular machine learning predicts all major clinical forms of drug cardiotoxicity

Polina Mamoshina^1,2*^, Alfonso Bueno-Orovio^1^, Blanca Rodriguez ^1^

^1^Department of Computer Science, University of Oxford, Oxford, United Kingdom

^2^Insilico Medicine Hong Kong Ltd, Pak Shek Kok, New Territories, Hong Kong

**SUPPORTING INFORMATION**

**Feature selection**

Analysis of the list of genes ranked as important by the two feature selection methods showed little to no intersection (Supporting Information Table 1). Conversely, consideration of Reactome pathways significantly improved the agreement (Supporting Information Table 2).

Both algorithms did not identify common genes for heart failure or coronary artery disorders, whereas they shared common Reactome pathways terms such as ‘G-alpha (i) signalling events’ for heart failure, and ‘Ub-specific processing proteases’ and ‘Regulation of TP53 Degradation’ for coronary artery disorders. Interestingly, G protein-coupled receptor transduction was selected as important by both methods for the prediction of cardiac disorder signs and symptoms (‘G alpha (q) signalling events’ and ‘G alpha (s) signalling events’) and heart failure (‘G-alpha (i) signalling events’). IGF1R and IGF1R-related signalling were also among the genes and Reactome terms selected by both methods for cardiac disorder signs and symptoms and for pericardial disorders. MAMLD1 gene, included in the Notch signalling pathways, was the only one ranked as important for predicting cardiac arrhythmias by both selection procedures.

**Consistency analysis**

To compare more robustly the two main approaches discussed in this work for cardiotoxicity prediction (chain of classifiers and a set of individual classifier), we performed consistency analysis. This was executed by repeating five times the split procedure of train and test sets described in the main manuscript (Section 2.4 Training, Validation and Testing Set Design of Methods). In this way, we created five different training and testing sets, and used them to first optimise the two approaches, and afterwards to test them on unseen data. Supplementary Figure 2 displays the distribution of AUC and MCC on validation and testing for the chain of random forest classifiers and the sets of individual random forest classifiers. The first approach outperforms the later on each of the five splits.

Our next part of the consistency analysis aimed to explore how the exclusion of individual cardiotoxicity forms impact the prediction accuracy. To do this, we trained and tested chain of classifiers to predict six cardiac disorders alternately removing cardiotoxicity forms starting from vascular disorders. Exclusion of labels reduced the accuracy of prediction of sequential labels on testing.

**Supporting Information figure and table legends:**

**Supporting Information Table 1.** Number of drugs with and without reported six forms of cardiotoxicities for four major pharmacological classes. CNS: central nervous system; CV: cardiovascular; S&S: signs and symptoms; dis: disorders.

**Supporting Information Table 2.** Common genes and pathways they are involved in between lists selected by two feature selection procedures

**Supporting Information Table 3**. Common pathways between lists selected by two feature selection procedures. Pathways which contain common genes between lists selected by two feature selection procedures are in bold.

**Supporting Information Table 4.** The performance of multi-label classification models trained on transcriptional profiles and molecular descriptors and fingerprints of drugs on the validation and the testing set.

**Supporting Information Table 5.** Predictions of the best model trained on transcriptional features, molecular descriptors and fingerprint and both. ANOVA test results for feature values and seven prediction groups. and number of samples per each group. All correct – drugs predicted correctly by the best predictor trained on transcriptional features, molecular descriptors and fingerprints and both. All false – drugs predicted incorrectly by the best predictor trained on transcriptional features, molecular descriptors and fingerprints and both. Dual&Desc false – drugs predicted correctly only by the best model trained on transcriptional features. Dual false – drugs predicted correctly by the best model trained on transcriptional features and molecular descriptors and fingerprints. Dual&Gene false – drugs predicted correctly only by the best predictor trained on molecular descriptors and fingerprints. Gene&Desc false – drugs predicted only by the best model trained on both transcriptional features and molecular descriptors and fingerprints. Gene false – drugs predicted correctly by the best predictor trained on only molecular descriptors and fingerprints on them combined with and transcriptional features.

**Supporting Information Figure 1.** Kernel density curves of distribution of AUC and MCC values on validation and testing for chain of classifiers (CC) and individual classifiers (BC). Mean values are displayed as dashed lines.

**Supporting Information Figure 2.** Levels of association measured between cardiotoxicity forms in the testing set in terms of Cohen’s Kappa.

**Supporting Information Figure 3.** Prediction of cardiotoxicity forms using the chain of classifiers with nested stacking (CC). AUC of best performing CC in safety drug prediction on validation (val) and testing sets (test) for each independent cardiac disorder in case of exclusion of one of the explored disorders. Mean values are displayed as dashed lines. S&S: signs and symptoms; dis: disorders.

**Supporting Information Figure 4.** Values of PSME1, HSPA8, PSME2, MDEC.11, VSPS28 and GAPDH features for drugs assigned to seven prediction groups according to the accuracy of the prediction. All correct – drugs predicted correctly by the best predictor trained on transcriptional features, molecular descriptors and fingerprints and both. All false – drugs predicted incorrectly by the best predictor trained on transcriptional features, molecular descriptors and fingerprints and both. Dual&Desc false – drugs predicted correctly only by the best model trained on transcriptional features. Dual false – drugs predicted correctly by the best model trained on transcriptional features and molecular descriptors and fingerprints. Dual&Gene false – drugs predicted correctly only by the best predictor trained on molecular descriptors and fingerprints. Gene&Desc false – drugs predicted only by the best model trained on both transcriptional features and molecular descriptors and fingerprints. Gene false – drugs predicted correctly by the best predictor trained on only molecular descriptors and fingerprints on them combined with and transcriptional features. Box plots display the median and interquartile range and whisker shows the lower and upper quartiles.

**Supporting Information Figure 5.** Values of nRotB, GFPT1, ATSc3, Kier2, DNAJC15 and SNCA features for drugs assigned to seven prediction groups according to the accuracy of the prediction. All correct – drugs predicted correctly by the best predictor trained on transcriptional features, molecular descriptors and fingerprints and both. All false – drugs predicted incorrectly by the best predictor trained on transcriptional features, molecular descriptors and fingerprints and both. Dual&Desc false – drugs predicted correctly only by the best model trained on transcriptional features. Dual false – drugs predicted correctly by the best model trained on transcriptional features and molecular descriptors and fingerprints. Dual&Gene false – drugs predicted correctly only by the best predictor trained on molecular descriptors and fingerprints. Gene&Desc false – drugs predicted only by the best model trained on both transcriptional features and molecular descriptors and fingerprints. Gene false – drugs predicted correctly by the best predictor trained on only molecular descriptors and fingerprints on them combined with and transcriptional features. Box plots display the median and interquartile range and whisker shows the lower and upper quartiles.

**Supporting Information Figure 6.** Values of PetitjeanNumber, nAromBond, MDEC.44, MDEC.14 and MDEC.13 features for drugs assigned to seven prediction groups according to the accuracy of the prediction. All correct – drugs predicted correctly by the best predictor trained on transcriptional features, molecular descriptors and fingerprints and both. All false – drugs predicted incorrectly by the best predictor trained on transcriptional features, molecular descriptors and fingerprints and both. Dual&Desc false – drugs predicted correctly only by the best model trained on transcriptional features. Dual false – drugs predicted correctly by the best model trained on transcriptional features and molecular descriptors and fingerprints. Dual&Gene false – drugs predicted correctly only by the best predictor trained on molecular descriptors and fingerprints. Gene&Desc false – drugs predicted only by the best model trained on both transcriptional features and molecular descriptors and fingerprints. Gene false – drugs predicted correctly by the best predictor trained on only molecular descriptors and fingerprints on them combined with and transcriptional features. Box plots display the median and interquartile range and whisker shows the lower and upper quartiles.

**Supporting Information Figure 7.** Values of tpsaEfficiency, topoShape, XLogP, WPATH, MLogP and MW features for drugs assigned to seven prediction groups according to the accuracy of the prediction. All correct – drugs predicted correctly by the best predictor trained on transcriptional features, molecular descriptors and fingerprints and both. All false – drugs predicted incorrectly by the best predictor trained on transcriptional features, molecular descriptors and fingerprints and both. Dual&Desc false – drugs predicted correctly only by the best model trained on transcriptional features. Dual false – drugs predicted correctly by the best model trained on transcriptional features and molecular descriptors and fingerprints. Dual&Gene false – drugs predicted correctly only by the best predictor trained on molecular descriptors and fingerprints. Gene&Desc false – drugs predicted only by the best model trained on both transcriptional features and molecular descriptors and fingerprints. Gene false – drugs predicted correctly by the best predictor trained on only molecular descriptors and fingerprints on them combined with and transcriptional features. Box plots display the median and interquartile range and whisker shows the lower and upper quartiles.

**Supporting Information Figure 8.** Values of AMR, IGF1R, NFKBIB, apol, TopoPSA and ALogp2 features for drugs assigned to seven prediction groups according to the accuracy of the prediction. All correct – drugs predicted correctly by the best predictor trained on transcriptional features, molecular descriptors and fingerprints and both. All false – drugs predicted incorrectly by the best predictor trained on transcriptional features, molecular descriptors and fingerprints and both. Dual&Desc false – drugs predicted correctly only by the best model trained on transcriptional features. Dual false – drugs predicted correctly by the best model trained on transcriptional features and molecular descriptors and fingerprints. Dual&Gene false – drugs predicted correctly only by the best predictor trained on molecular descriptors and fingerprints. Gene&Desc false – drugs predicted only by the best model trained on both transcriptional features and molecular descriptors and fingerprints. Gene false – drugs predicted correctly by the best predictor trained on only molecular descriptors and fingerprints on them combined with and transcriptional features. Box plots display the median and interquartile range and whisker shows the lower and upper quartiles.

**Tables and Figures**

Supplementary Table 1.

| Pharmacological class | Reports of cardiac toxicity | Cardiac dis S&S | Cardiac arrhythmias | Coronary artery dis | Heart failures | Myocardial dis | Pericardial dis |
| --- | --- | --- | --- | --- | --- | --- | --- |
| CNS | yes | 164 | 129 | 102 | 49 | 16 | 9 |
|  | no | 374 | 409 | 436 | 489 | 522 | 529 |
| CV | yes | 61 | 34 | 36 | 24 | 2 | 1 |
|  | no | 99 | 126 | 124 | 136 | 158 | 159 |
| Anti-inflammatory | yes | 24 | 11 | 10 | 4 | 2 | 1 |
|  | no | 59 | 72 | 73 | 79 | 81 | 82 |
| Antineoplastic | yes | 46 | 25 | 23 | 13 | 3 | 7 |
|  | no | 47 | 68 | 70 | 80 | 90 | 86 |

Supplementary Table 2.

| Gene Symbol | Gene Name | Pathways |
| --- | --- | --- |
| Cardiac disorder signs and symptoms | | |
| DECR1 | 2,4-Dienoyl-CoA Reductase 1 | mitochondrial fatty acid beta-oxidation of unsaturated fatty acids |
| IGF1R | Insulin Like Growth Factor 1 Receptor | SHC-related events triggered by IGF1R; Signaling by Type 1 Insulin-like Growth Factor 1 Receptor (IGF1R); IRS-related events triggered by IGF1R |
| Cardiac arrhythmias | | |
| MAMLD1 | Mastermind Like Domain Containing 1 | Pre-NOTCH Transcription and Translation; Constitutive Signaling by NOTCH1 PEST Domain Mutants; Notch-HLH transcription pathway; NOTCH2 intracellular domain regulates transcription; RUNX3 regulates NOTCH signaling; Constitutive Signaling by NOTCH1 HD+PEST Domain Mutants; NOTCH1 Intracellular Domain Regulates Transcription; Pre-NOTCH Transcription and Translation |
| Heart failures | | |
| NA | | |
| Coronary artery disorders | | |
| NA | | |
| Pericardial disorder | | |
| IGF1R | Insulin Like Growth Factor 1 Receptor | SHC-related events triggered by IGF1R; Signaling by Type 1 Insulin-like Growth Factor 1 Receptor (IGF1R); IRS-related events triggered by IGF1R |
| IER3 | Immediate Early Response 3 | PI5P, PP2A and IER3 Regulate PI3K/AKT Signaling |
| GATA3 | GATA Binding Protein 3 | Interleukin-4 and 13 signaling; RUNX1 regulates transcription of genes involved in differentiation of HSCs; Ub-specific processing proteases; Factors involved in megakaryocyte development and platelet production |
| NUCB2 | Nucleobindin 2 | NA |
| LPGAT1 | Lysophosphatidylglycerol Acyltransferase 1 | Acyl chain remodelling of PG |
| SH3BP5 | SH3 Domain Binding Protein 5 | NA |
| Myocardial disorders | | |
| HMGCS1 | 3-Hydroxy-3-Methylglutaryl-CoA Synthase 1 | Cholesterol biosynthesis; PPARA activates gene expression; Activation of gene expression by SREBF (SREBP) |

Supplementary Table 3.

| Pathways |
| --- |
| Cardiac disorder signs and symptoms |
| G alpha (q) signalling events |
| FCERI mediated NF-kB activation |
| Downstream TCR signaling |
| Activation of NF-kappaB in B cells |
| CLEC7A (Dectin-1) signaling |
| Ub-specific processing proteases |
| RUNX1 regulates transcription of genes involved in differentiation of HSCs |
| **mitochondrial fatty acid beta-oxidation of unsaturated fatty acids** |
| HATs acetylate histones |
| **SHC-related events triggered by IGF1R** |
| **Signaling by Type 1 Insulin-like Growth Factor 1 Receptor (IGF1R)** |
| **IRS-related events triggered by IGF1R** |
| G alpha (s) signalling events |
| Neutrophil degranulation |
| Cardiac arrhythmias |
| **Pre-NOTCH Transcription and Translation** |
| **Constitutive Signaling by NOTCH1 PEST Domain Mutants** |
| **Notch-HLH transcription pathway** |
| **NOTCH2 intracellular domain regulates transcription** |
| **RUNX3 regulates NOTCH signaling** |
| **Constitutive Signaling by NOTCH1 HD+PEST Domain Mutants** |
| **NOTCH1 Intracellular Domain Regulates Transcription** |
| mRNA Splicing - Major Pathway |
| Separation of Sister Chromatids |
| ER-Phagosome pathway |
| AUF1 (hnRNP D0) binds and destabilizes mRNA |
| IKK complex recruitment mediated by RIP1 |
| MHC class II antigen presentation |
| Neutrophil degranulation |
| Collagen degradation |
| Degradation of the extracellular matrix |
| Clathrin-mediated endocytosis |
| Interleukin-4 and 13 signaling |
| Heart failures |
| G alpha (i) signalling events |
| Coronary artery disorders |
| Ub-specific processing proteases |
| Regulation of TP53 Degradation |
| Pericardial disorders |
| Orc1 removal from chromatin |
| Activation of ATR in response to replication stress |
| Activation of the pre-replicative complex |
| **Interleukin-4 and 13 signaling** |
| **Ub-specific processing proteases** |
| **RUNX1 regulates transcription of genes involved in differentiation of HSCs** |
| Factors involved in megakaryocyte development and platelet production |
| Interleukin-3, 5 and GM-CSF signaling |
| Platelet sensitization by LDL |
| **Acyl chain remodelling of PG** |
| Post-translational protein phosphorylation |
| **Regulation of Insulin-like Growth Factor (IGF) transport and uptake by Insulin-like Growth Factor Binding Proteins (IGFBPs)"** |
| **PI5P, PP2A and IER3 Regulate PI3K/AKT Signaling** |
| **SHC-related events triggered by IGF1R** |
| **Signaling by Type 1 Insulin-like Growth Factor 1 Receptor (IGF1R)** |
| **IRS-related events triggered by IGF1R** |
| RHO GTPases Activate Formins |
| Myocardial disorders |
| **Activation of gene expression by SREBF (SREBP)** |
| **Cholesterol biosynthesis** |
| **PPARA activates gene expression** |

Supplementary Table 4.

Provided in separate file: “data sheet 2.xls”

Supplementary Table 5.

Provided in separate file: “data sheet 3.xlsx”.

Supplementary Figure 1


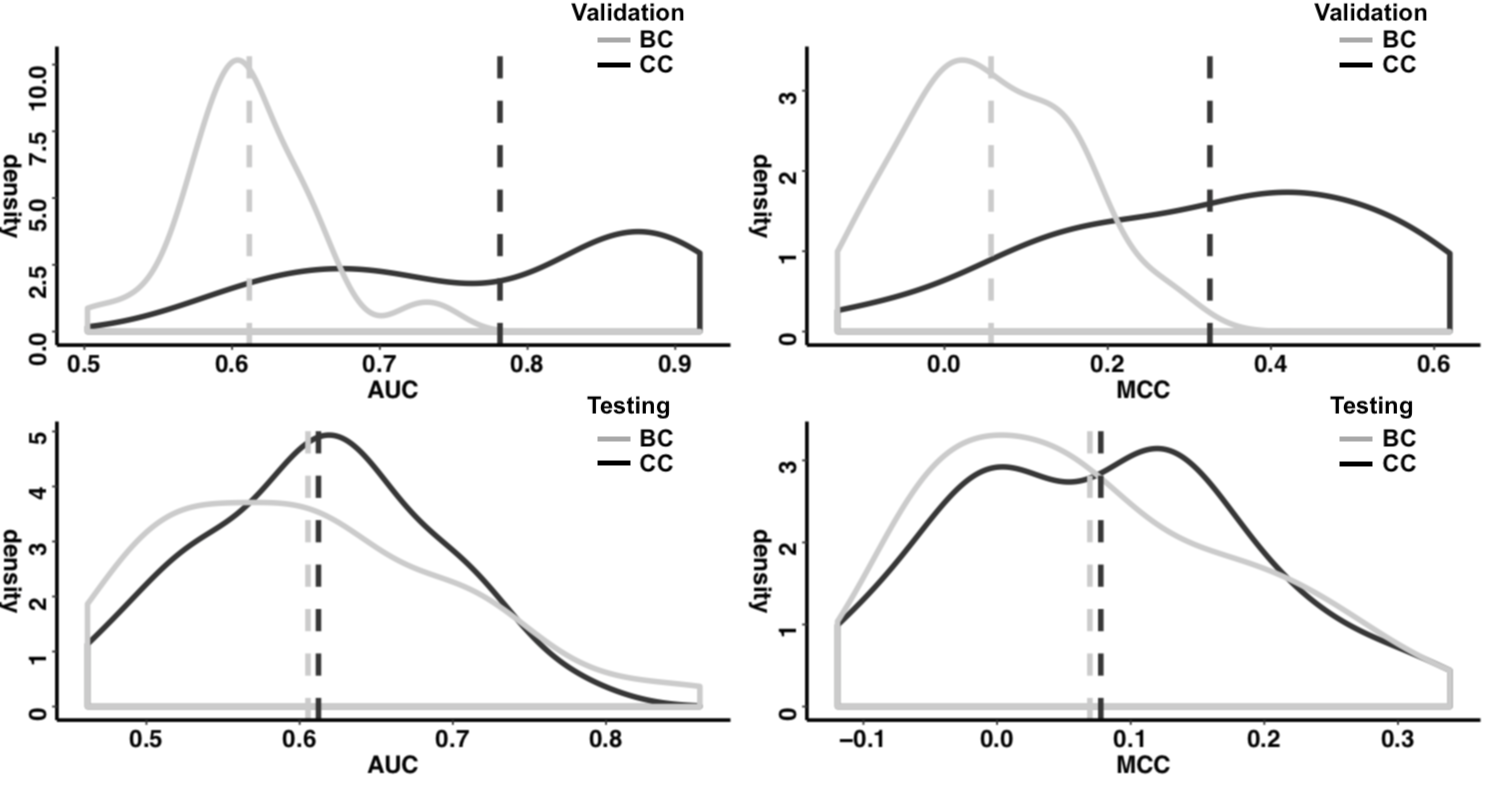


Supplementary Figure 2

Supplementary Figure 3


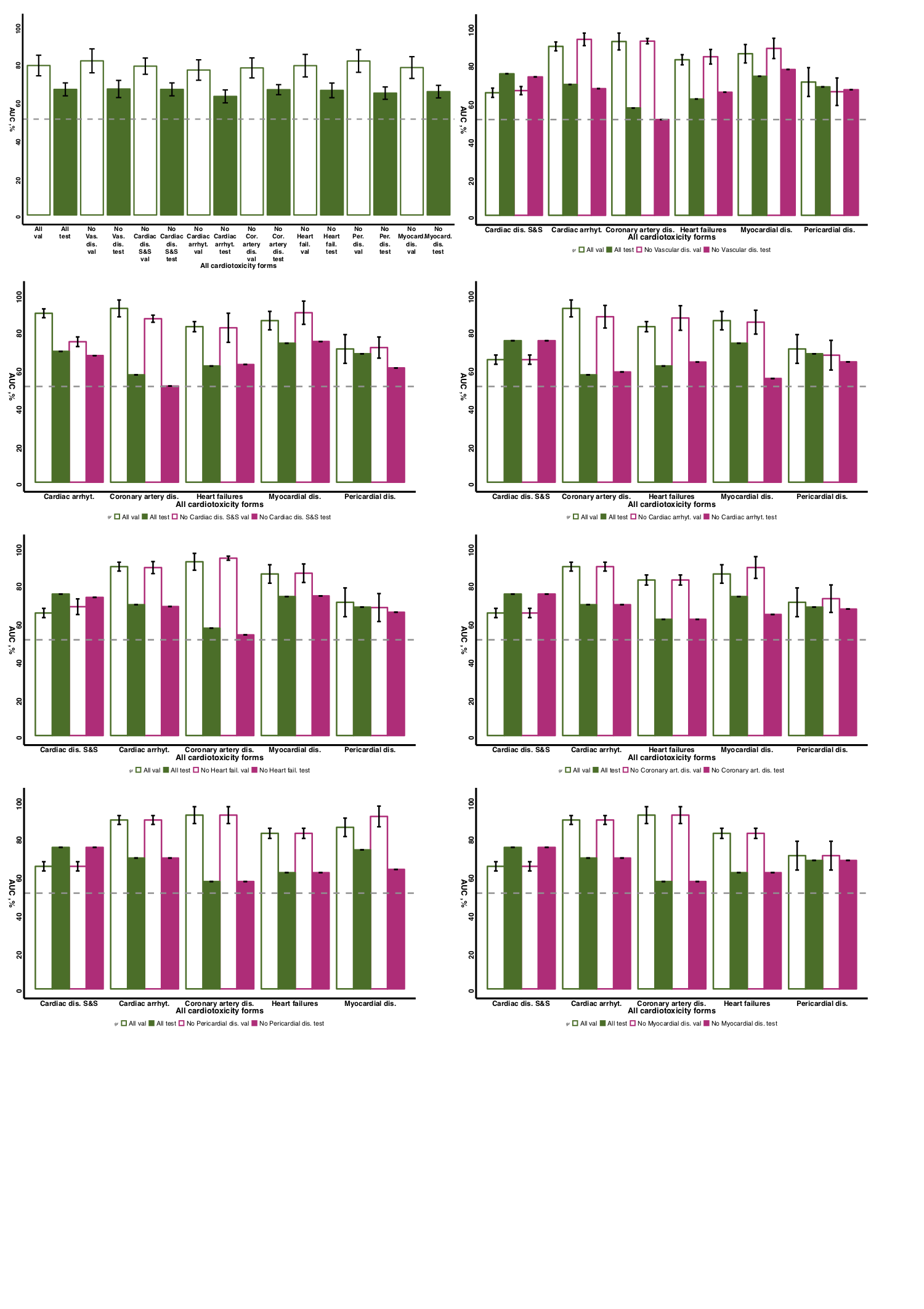


Supplementary Figure 4


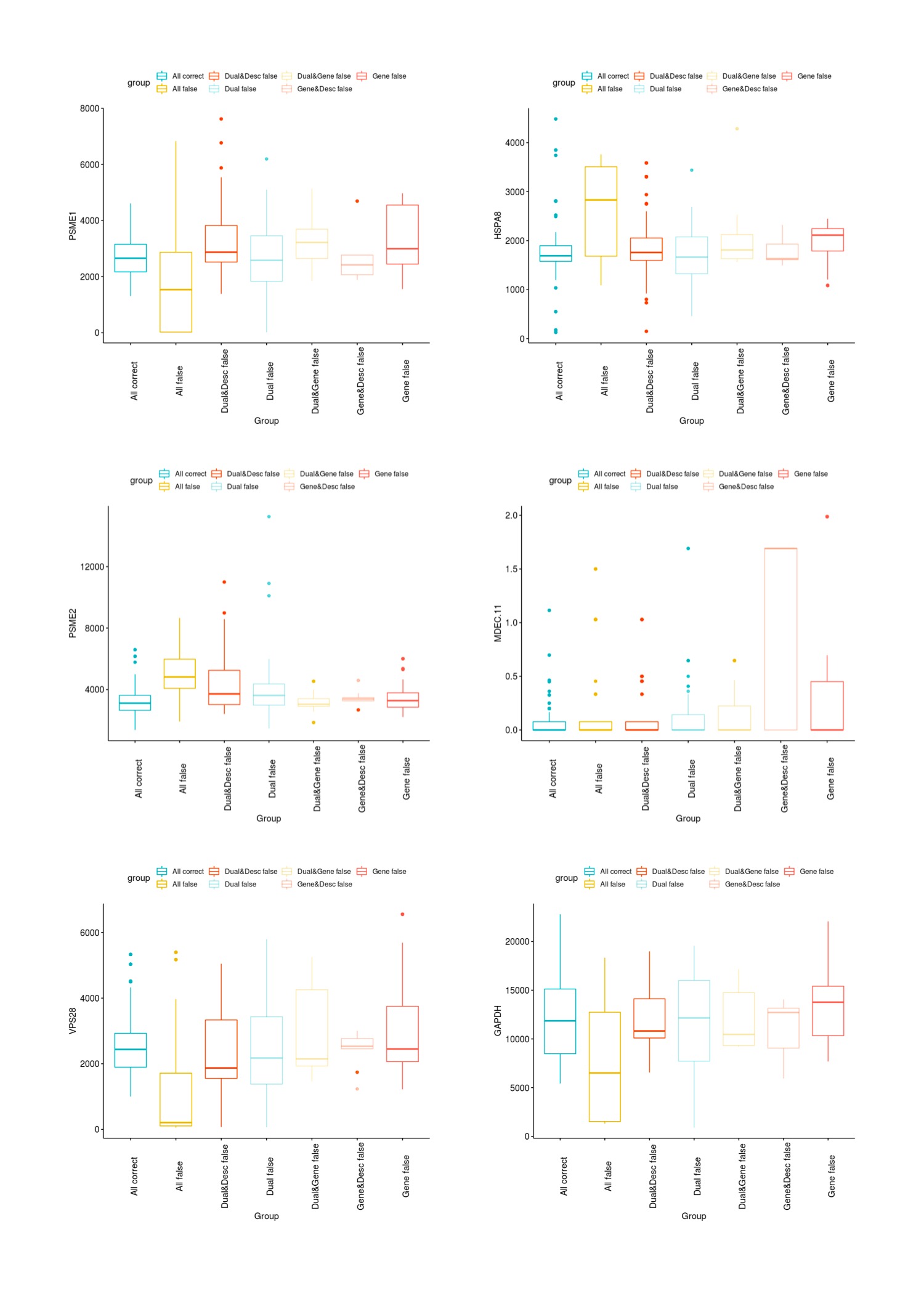


Supplementary Figure 5


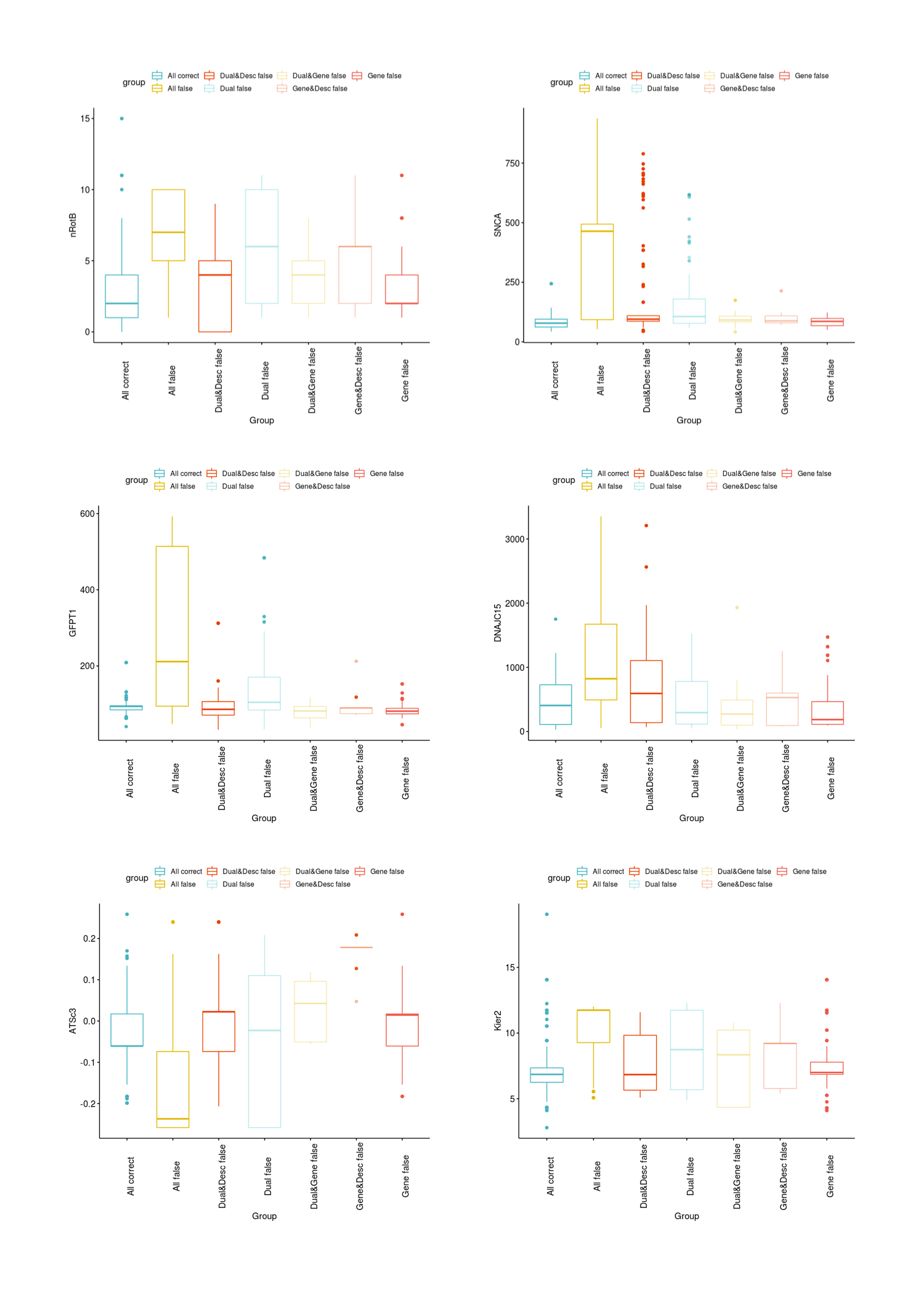


Supplementary Figure 6


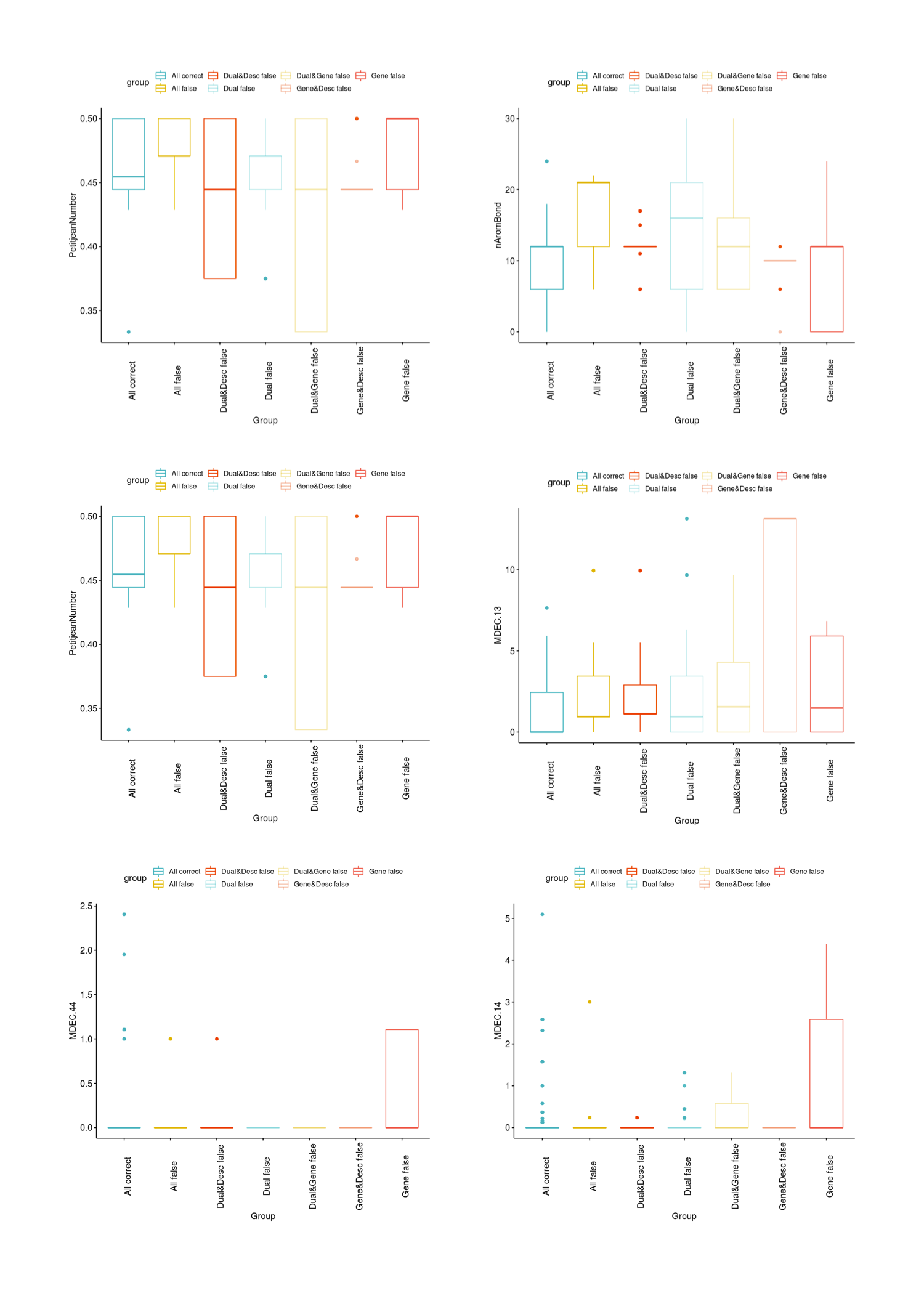


Supplementary Figure 7


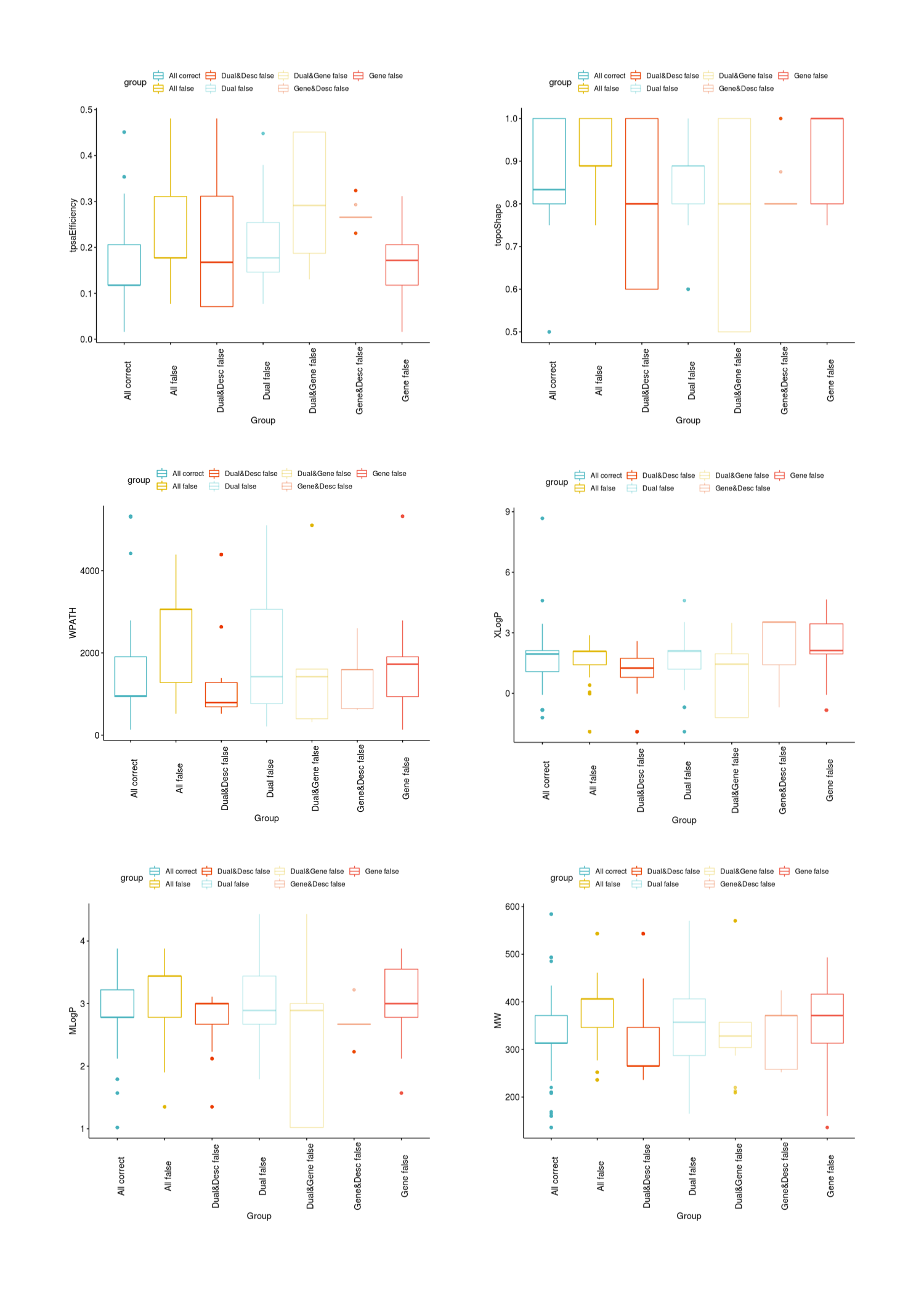


Supplementary Figure 8


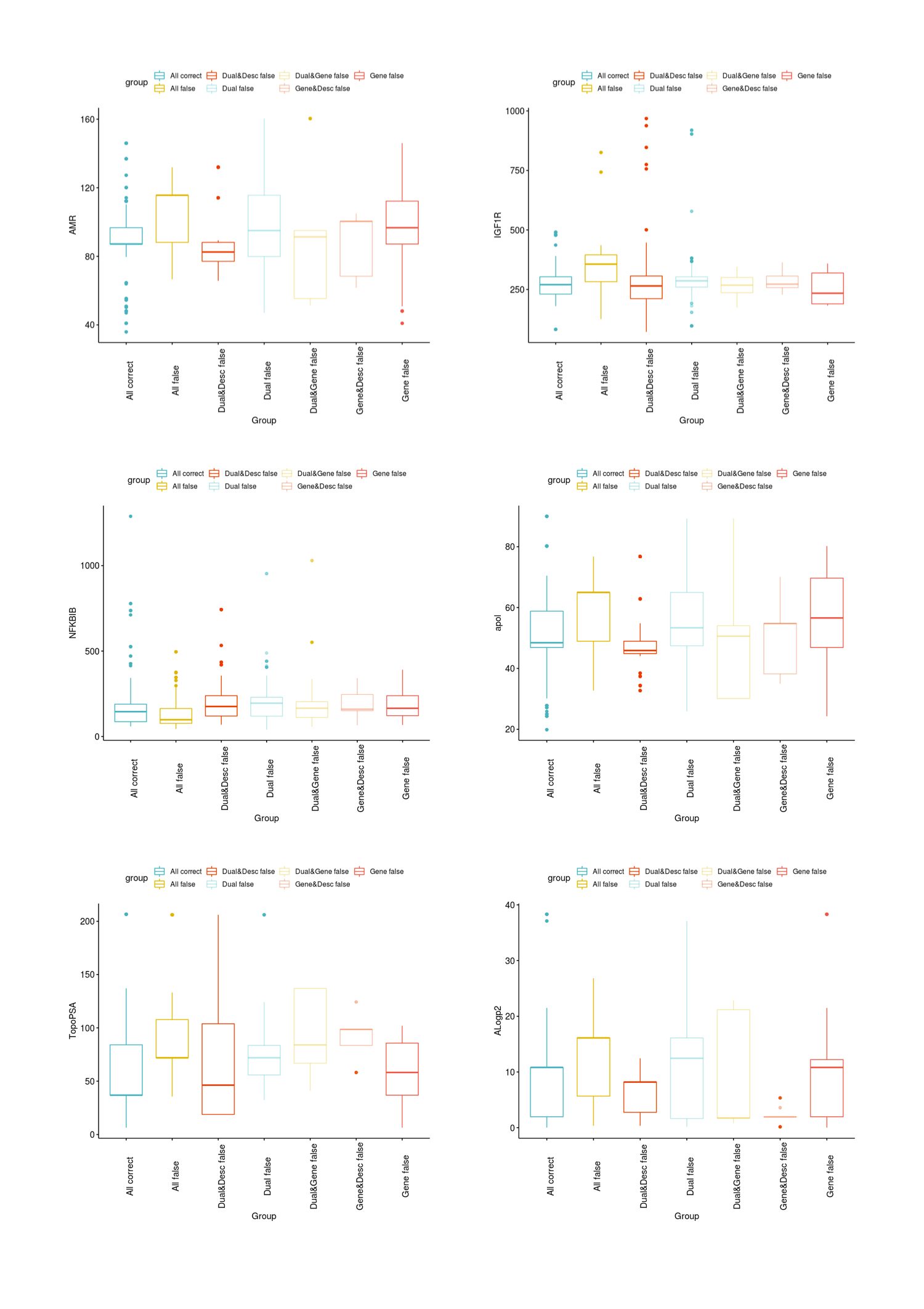

Supplement: Supplementary file 1 [file DataSheet_1.docx]
